# Hospital and Institutional News

**Published:** 1918-07-13

**Authors:** 


### HOSPITAL AND INSTITUTIONAL NEWS.

### SIR FRANCIS LLOYD ON VOLUNTARY EFFORT.

Hospitals must appreciate the surgical contrivances which are being made by the million at the workrooms in Mulberry Walk, Chelsea, of the Surgical Requisites Association, which is the orthopædic branch of Queen Mary's Needlework Guild. Many of the apparatus, which include splints of all descriptions, peg legs, surgical boots, gloves, baths, and bandages, have been invented by members of the Association. Where necessary the appliances are made to measure. Opening an extension of the premises last week, Lieutenant-General Sir Francis Lloyd admired the apparatus made voluntarily by so many industrious ladies. Whenever any hospital wanted a man fitted for any sort of appliance it was able to send him to Chelsea. The sad thing about institutions such as theirs, he said, was that they seemed to be standing on the brink of a precipice, inasmuch as the Government could neither make up its mind to take them over nor to assist them financially. He trusted, however, that the Ministry of Pensions would see that their work did not suffer for want of money. The voluntary workers of Great Britain by their wonderful efforts had undoubtedly put the State to shame. He believed that the huge voluntary organisation which existed in England to-day could not be achieved in any other country.

### HOSPITAL SHIPS SEARCHED BY GERMANS.

It came out during question-time on Monday last in the House of Commons that, on several occasions, German submarines have exercised their right, under the Hague Convention, to stop and search hospital ships in order to see that they are complying with the terms of the international agreement. It goes without saying that no cause of offence has been found, and Dr. Macnamara informed the House that in no case has this country ever broken the letter or spirit of the Hague Convention regarding hospital ships, and the same statement is believed to be true as regards our Allies. In the last dastardly outrage the Huns appear to have executed the prisoner before the trial and after every vestige of material evidence had been destroyed. It is probable that submarines do not care to linger over their fell work, and that any semblance of search would mean for them dangerous delay. Mr. Bonar Law told the House of Commons that "all possible means of safeguarding our hospital ships are at present under consideration in consultation with our Allies." At present it seems almost safer for these ships to proceed on their voyages without any of the lights or signs that should provide them with immunity from attack.

### THE AMERICAN HOSPITAL NEAR SOUTHAMPTON.

Further details are now available of the great American Military Hospital at Sarisbury Court, near Southampton, where nearly 3,000 wounded will be accommodated. The old manor-house will be the centre of ten acres of huts; another ten acres, the old vegetable garden, will be intensively cultivated, so that the hospital may largely supply itself with garden and indeed with farm and dairy produce. The whole estate comprises 186 acres. Captain F. Harper Sibley, who has been in charge of American Red Cross work at Southampton since April, is supervising the construction. Some of the first 400 beds will be placed in "Bossareau" huts, whose double roofs and long windows should secure an even temperature. The ambulances which will meet the hospital ships on their arrival at Southampton will carry the wounded to a pleasant estate, where the convalescents will find many walks within the grounds, where the woods and meadows slope southwards to the shore.

### AN INFIRMARY CHAPLAIN'S "ESPRIT DE CORPS."

That the Poor-Law infirmary is developing an *esprit de corps* in the active members of its staff is one sign of the great improvement which has taken place of late years. Thus the Rev. Leonard Joyce's recent letter to the *Pall Mall Gazette* is a landmark on the road to their complete separation from the Poor Law. Mr. Joyce, the acting chaplain to Lambeth Infirmary, who is at pains to show that "no stigma of pauperism attaches to patients in infirmaries," proceeds: "In the one where I am working there are patients who pay considerable sums a week, and quite a number, I believe, who pay lesser sums. I doubt whether there are as many paying patients in general hospitals as in infirmaries, for in the latter the Guardians exact payment wherever circumstances admit of it. Moreover, infirmaries are supported by rates, to which all contribute, whereas hospitals are supported out of alms. Military hospitals are financed by taxes. So that infirmaries and military hospitals are institutions to which all perforce pay their share and have a right to treatment, while general hospitals are almost entirely charity institutions." We question whether Mr. Joyce is right in asserting that there are fewer paying patients in general hospitals than in infirmaries. The latter are still, technically, bound to admit "the destitute" only, and that in practice they admit ratepayers has not yet sufficed to remove the sensation of being stamped as a pauper on admission in the mind of the public at large. Many people know the facts, but so long as the technicality perpetuates the old prejudice, so long will the facts be misunderstood. That is why the association of the infirmary with the Poor Law should be theoretically as well as practically abolished.

### THE RIGHT TO INTRODUCE VISITORS WITHOUT PERMISSION.

The dissatisfaction already expressed by the committee of the Cardiff Mental Hospital and by Colonel Goodall, the C.O., with Colonel Lynn Thomas, Deputy-Inspector of Military Orthopædics, for introducing various visitors to the institution without warning to either authority, has again been aroused. Since the committee's protests in regard to the visits of King Manoel and other prominent persons, which Colonel Lynn Thomas arranged without notice, a similar visit of Mr. John Hodge, the Minister of Pensions, has been complained of. It was carried by resolution that "no visits to the hospital, other than those of the Board of Control, Authorities of the War Office, and of the Western Command, be permitted without the sanction of the committee, or of Colonel Goodall." The committee feels affronted by these incidents, and Colonel Goodall points out that Colonel Lynn Thomas's right of entry is limited to the orthopædic section, and that he has no right to introduce other persons without the usual permission.

### THE PRICE OF TAKING SCHOOLS FOR HOSPITALS.

Considerable dissatisfaction is expressed at the buildings provided by the London County Council for the education of over 7,000 children whose schools have been taken for military hospitals. They are five in number, and all are described as "good type schools." The displaced children were housed in two other schools, where they only receive tuition in the summer three and a half hours a day, and in the winter three hours. Investigations show that the children leaving these schools cannot compete on equal or favourable terms with those from neighbouring schools, whose school-hours have not been curtailed, and are frequently unable to pass simple proficiency tests for employment, which ordinarily should be within their reach. The Camberwell Borough Council, which has been approached by an influential deputation, concludes that the London County Council has had ample time to provide proper substitutes for the commandeered schools and has decided to interview Sir Robert Blair, the Chief Education Officer for London. If suitable buildings have been found, how comes it that the hours of teaching have been so much reduced?

### THE FIRE AT CARDIFF.

The new Welsh Medical School at Cardiff, which has suffered many delays, official and other, was the scene of a recent fire when one of the laboratories on the top floor was badly damaged. The building is not finished, but unfortunately the laboratory had been equipped, and it was in the laboratory that the fire originated. The enthusiasts at Cardiff deserve public sympathy, and probably this fire will dishearten them less than some of their diplomatic difficulties.

### IMPROVEMENTS AT GLOUCESTER ROYAL INFIRMARY.

Among the numerous hospitals which are setting up electro-therapeutic or massage departments is the Gloucester Royal Infirmary. But the progress of the work is hampered by war restrictions on every hand. A permit is necessary for the smallest machine: in fact a permit is generally necessary for a permit! However, the drainage has been installed, the interior decorations carried out, and the electric and hot-water installations are being constructed. A recent improvement is the disinfector, which has been established, with the advice of Mr. Sisson, at a cost of £185. It performs a double service by its one work, which obviates the old arrangement whereby large articles requiring disinfection had to be sent to Over Hospital. This inconvenience is now a thing of the past.

### THE BUTE FAMILY AND KING EDWARD VII. HOSPITAL, CARDIFF.

At the recent meeting of the Needlework and Linen Guilds at the above hospital, when the Marchioness of Bute presided, Colonel Bruce-Vaughan, the vice-chairman, gave a brief historical sketch of the connection of her family with the institution. He believed he had the pleasure of introducing her to Cardiff on the first occasion of a public visit after her marriage, when she visited the hospital. About eighty-four years ago, an Eisteddfod was held at Cardiff Castle, over which the present Marquis of Bute's grandfather presided. The £300 collected on that occasion to establish a hospital in Cardiff was the nucleus of the good work which had been done since. The foundation-stone was laid in 1837. About 1880 the father of the present Marquis gave the site of the present hospital. Ever since the foundation-stone was laid, three years later, the family connection had been kept up. Much work yet remained. The waiting-list still comprised 1,119 names, but at the end of eighteen months he hoped that the beds would be increased from 329 to 500, and that the Lord Mayor's scheme for raising £34,000 a year would be successful.

### AN EXAMPLE TO BE FOLLOWED UP AT YORK.

We have had to postpone until this week an account of the healthy growth of the York Workpeople's Hospital Fund, which not only reached practically £900, but celebrated its seventeenth birthday by a presentation to Mr. Arthur Wilkinson, who has been its active honorary secretary since the foundation of the fund in 1901. The presentation took the form of a silver tea-service and a pocket-wallet. On the teapot was inscribed: "Presented to Councillor Arthur Wilkinson as a mark of appreciation of over seventeen years' most devoted and self-sacrificing labours as honorary secretary of the York Workpeople's Hospital Fund." A silver-mounted umbrella was also presented to Mrs. Wilkinson, the mother of Councillor Wilkinson, and to Miss Wilkinson, his sister, in recognition of their assistance in his work. The fund has now raised £22,000 in all, and this year again showed an increase. We hope, as we have suggested before, that the success of this fund may lead the way to the organisation of sectional subscriptions from other classes, and that the beginning made in the case of the farmers may be followed up, so that each class served by the hospitals may have its own fund and its own organisation.

### A UNANIMOUS RESOLUTION.

At a meeting of the Board of Management of the Royal West Sussex Hospital, Chichester, held on July 9, on the eve of the departure of the secretary, Mr. Tom B. Harrison, to his new post at Buxton, the following resolution was passed: "That in tendering Mr. Harrison the best wishes of the Board of Management on his departure to a higher sphere of responsibility in the Hospital world, the Board wish to place on record their very high appreciation of those qualities which have made his tenure of office in this hospital so successful. Always courteous, obliging, and indefatigable in his work, he has earned the highest esteem of all he has come in contact with, both Board and patients. During the very heavy strain that was thrown on this institution during the reconstruction his work was invaluable, and it was chiefly through his devotion to the work, and that of the Matron, that half of the buildings were kept open for patients during the whole time."

### A NEW SCALE OF FEES FOR DOCTORS.

AN Army Council instruction has been issued which states that, in consequence of the increased cost of travelling, the present scale of fees for civilian medical practitioners will be cancelled, and a new scale will be published in its place.

### THE CHANCE OF A LIFETIME FOR MEDICAL WOMEN.

MISS MAJOR, the Administrative Officer of King's College for Women, speaking at the University last week, said that the women students, in conjunction with those of similar bodies, were helping to endow a bed in the Elizabeth Garrett Anderson Hospital as a memorial to that pioneer of medical women. Despite discouragement in her young days, Dr. Anderson pursued the study of medicine and encouraged it among her sex. In consequence there were to-day hospitals at the Front which were entirely staffed by women. One or two women from King's College would shortly become dressers in a front-line hospital. Viscount Hambleden predicted great opportunities for women medical students, because so many male medical students and so many qualified medical men had fallen in the war. In fact, he foresaw a serious shortage of surgeons and physicians both in our great hospitals and in private practice.

### AN AMERICAN UNIT FOR PALESTINE.

THE American Zionist Medical Unit, which arrived in this country on June 24, paused in London to make final arrangements before leaving for Palestine. The party, which is in charge of Mr. Lewin Epstein, consists of forty-four persons, including fifteen physicians and dentists, twenty nurses, two sanitary specialists, and social workers and administrators. The unit's equipment of over 400 tons weight, comprising a 100-bed hospital, $x$-ray apparatus, drugs, instruments, ambulances, and clothes for the destitute (Jewish and non-Jewish) among which it will work, was shipped from New York direct to Egypt.

### SALARIES FOR VACCINATION OFFICERS.

THE Southwark Guardians, who have never pressed the Vaccination Acts in their district, have had forcibly brought to their notice the great decline there has been in the number of public vaccinations during the past few years. One of their vaccination officers, who had been in their service for over twenty-five years, applied for a fixed salary. He pointed out that his fees in 1914 amounted to £140, but these fell last year to £102, whilst in the first quarter of this year they were merely £21. Various times his fees have been increased so that his remuneration might be kept at a proper level, but reduced population and less vaccinations, largely in consequence of the Vaccination Act, 1907, which gave greater facilities for exemption, have brought about reduction in the fees. The Guardians have, therefore, decided to ask the Local Government Board to permit them to pay the officers a salary of £160 a year, exclusive of postage. In adjoining parishes the Local Government Board before the war assented to fixed salaries being given to vaccination officers, and there is little doubt that they will agree to it in the case of Southwark.

### A NEW MUNICIPAL POST.

IT is the custom nowadays to have welfare workers in factories, but the Birmingham Corporation is the first to have a welfare superintendent for the benefit of the women employed by the municipality. Since the war crowds of women and girls have been employed by municipal authorities, especially those having trading concerns. The Corporation has appointed Mrs. D. Hamilton Shaw as superintendent—a position she now holds in a large factory in the Midlands. At the outset she will confine her attention to the women and girls engaged in the old Council House and at the Food Offices, but the Corporation reports that later on other departments may desire the advantages of her services.

### AN ATLAS FOR THE BLIND.

ONE of the latest productions of the National Institute for the Blind, Great Portland Street, W., is a geographical and industrial atlas of the British Isles. The resources of the Institute make it possible to sell this important work to blind students at half-a-crown a copy. The book is the outcome of years of experimenting and improvement upon old maps for blind people; the difficulty of providing a map that can be studied by means of the finger-tips has been great indeed, but, largely owing to the efforts of Mr. H. M. Taylor, F.R.S., of Cambridge, himself sightless, the Institute can say that, though perfection has not perhaps been attained, the present series of maps is a distinct advance on anything that has gone before. The work is very complete, and follows to the smallest detail the ordinary printed atlas. Towns, rivers, boundaries, coast-lines, mountain ranges, and railways are all distinctly marked. Each one of these has a number marked against it which corresponds with a key which is on the opposite page to the map. The volume is a large one, containing 222 pages, twenty-one of which are maps, and is bound in cloth boards. A more suitable present for a blind friend it would be difficult to find.

### WHERE TO KEEP THE MILK.

Hospital managers will have had during the last week or so another worry added to the ever-extending list, one small in itself, but sufficiently perplexing to cause the taking up of a substantial amount of time in the solving of a new difficulty. The milk companies, owing to the shortage of churns and the difficulty in replacing worn-out ones, will not now leave the churn with the day's supply of milk, therefore hospitals have to provide receptacles for storage. New crocks and cans of an important size are not too easily obtained, and people who supply them will no doubt be called upon to produce not one or two, but many for this new purpose, and will not be able to do so all at once. Improvisation will be the order of the day to commence with, but a hospital of, say, 150 beds would require about fifty gallons of milk a day, and fifty gallons of milk will not go into a few jugs or tins. At one hospital in London it is proposed to use for the purpose a disused, full-length, porcelain bath, and, failing nothing better, this will do quite well to go on with; its chief bad points will be the fact that a cover of some sort must be provided, and one of wood, the most handy material for rapid construction, would be very heavy to take on and off. The best receptacle would be one that would not take up so much floor space, would be easily covered, and could be drained off for cleaning purposes from the bottom.

### THE ARMY PHARMACEUTICAL SERVICE: A BELATED CONCESSION.

After much representation and repeated interviews, the Army Council has notified the Council of the Pharmaceutical Society that in future all qualified pharmacists, who make the request on joining the Army, will be posted to the R.A.M.C., and, as far as possible, use will be made of their technical knowledge. No mention is made of any rank being granted to pharmacists as such, but as the value of their work becomes more widely known some rank, such as that of staff-sergeant, will no doubt be granted. Now that medical men obtain a full lieutenancy on joining the R.A.M.C. there is a good opportunity to give the " one pip " distinction to pharmacists on joining the Colours.

### INCREASED SALARY FOR POOR-LAW DISPENSERS

The Local Government Board has notified the Public Pharmacists' Association, in reply to a petition on the subject, that the Board is willing to allow a maximum salary of £200 per annum to dispensers in the Metropolitan area of the Poor-Law Service without the previous stipulation that " any increase in salary over the 'scale' one of £180 must only be granted by Boards of Guardians after long service or in exceptional circumstances." Whilst the adoption of this new scale is not obligatory on the part of the Boards of Guardians, it is probable that applications from their dispensers to be placed on the new scale will be favourably considered. But it is to be regretted that the Local Government Board has not agreed to increase the commencing salary, which, at £120 per annum, is very low, having regard to the status of dispensing.

### THE AMUSEMENTS OF EXILE.

Probably it is the enterprise shown by the members of the prisoners' camp at Ruhleben which has made it the best known in this country. A further and excellent account of the arts by which time was beguiled, health maintained, and the fiend *ennui* kept at a distance is contained in the June number of *The British Prisoner of War*. The writer is Mr. Jack Cameron, who speaks from three years' experience, and his jolly account will do good service if it reminds us that imprisonment, like exile, is a severe punishment, and that, with reference to English prisoners, hard labour has been described, with the sting that is the heart of humour, as " the only alleviation of the prisoner's lot." There are a few more " letters from Turkey " in the same journal, which does something, in spite of the difficulties, to lift the veil which makes a prisoner's life in Germany not only one of privation, but of isolation. We miss with regret any account of the life of British prisoners in Austria. Information from apparently excellent sources has reached us that British prisoners, officers, at least, have been kindly treated, allowed to make excursions, well fed, and comparatively free. If this is so, an account from Austria would be welcome. Is it beyond the power of *The British Prisoner of War* to reveal the facts?

### THIS WEEK'S DRUG MARKET.

The strong demand for quinine continues, and the price tendency is upwards; were it not for the fact that quinine is more or less under control, there can be little doubt that prices would be much higher than they are at present—in fact, the statistical position would seem to justify higher prices. Morphine is substantially dearer; the advance, which was long overdue, is in consequence of the high cost of opium. A rise in the price of rectified spirit has been followed by an advance in quotations for tinctures and other spirituous preparations. Aspirin tends higher in price, as also does salol. A fair business has been done in star aniseed oil at higher prices. Phenacetin and phenazone still have a slightly downward tendency in price. Business has been done in Curaçoa aloes at fully maintained rates. Cassia fistula is dearer. A good business has been done in honey at rather lower rates.

To obtain **The HOSPITAL**

